# Comparative analysis of differentially abundant proteins between high and low intramuscular fat content groups in donkeys

**DOI:** 10.3389/fvets.2022.951168

**Published:** 2022-07-27

**Authors:** Xiaofan Tan, Yu He, Yanchun Qin, Zhiwei Yan, Jing Chen, Ruixue Zhao, Shenglan Zhou, David M. Irwin, Bojiang Li, Shuyi Zhang

**Affiliations:** ^1^Department of Animal Genetics, Breeding and Reproduction, College of Animal Science and Veterinary Medicine, Shenyang Agricultural University, Shenyang, China; ^2^Department of Laboratory Medicine and Pathobiology, University of Toronto, Toronto, ON, Canada

**Keywords:** donkey, IMF, proteomics, differentially abundant proteins, functional analysis

## Abstract

Intramuscular fat (IMF) is an important regulator that determines meat quality, and its content is closely related to flavor, tenderness, and juiciness. Many studies have used quantitative proteomic analysis to identify proteins associated with meat quality traits in livestock, however, the potential candidate proteins that influence IMF in donkey muscle are not fully understood. In this study, we performed quantitative proteomic analysis, with tandem-mass-tagged (TMT) labeling, with samples from the longissimus dorsi (LD) muscle of the donkey. A total of 585,555 spectra were identified from the six muscle samples used in this study. In total, 20,583 peptides were detected, including 15,279 unique peptides, and 2,540 proteins were identified. We analyzed differentially abundant proteins (DAPs) between LD muscles of donkeys with high (H) and low (L) IMF content. We identified 30 DAPs between the H and L IMF content groups, of which 17 were upregulated and 13 downregulated in the H IMF group. Gene Ontology (GO) and Kyoto Encyclopedia of Genes and Genomes (KEGG) functional enrichment analysis of these DAPs revealed many GO terms (e.g., bone morphogenetic protein (BMP) receptor binding) and pathways (e.g., Wnt signaling pathway and Hippo signaling pathway) involved in lipid metabolism and adipogenesis. The construction of protein–protein interaction networks identified 16 DAPs involved in these networks. Our data provide a basis for future investigations into candidate proteins involved in IMF deposition and potential new approaches to improve meat quality in the donkey.

## Introduction

Donkeys are domesticated animals that belong to the horse family (1). They are one of the most important livestock animals in many countries in Africa and the Middle East, which are mainly used for farming or other work on large farms (2, 3). With the increase of interest in donkey breeding, it is increasingly being used as a milk and meat-producing animal (4, 5). Donkey milk has been shown to be the best substitute for human milk for children who are allergic to milk proteins (6). Donkey meat is characterized by high-quality protein, vitamins, and minerals, which is the preferred meat for many consumers due to its high protein content (5, 7, 8). According to a previous study, 100 g of donkey meat contains 22.8 g of protein and 2.02 g of fat (2, 9, 10). With improvements in living standards, consumers are becoming more concerned about the quality of livestock meat. In recent years, donkey meat consumption has increased in many countries, including China and Italy, and has undoubtedly become one of the livestock meat choices (6, 11, 12).

Intramuscular fat (IMF), also referred to as marbling, is the amount of fat that accumulates between muscle fibers or inside muscle cells (13). Its main components are phospholipids and triglycerides (14, 15). A previous study reported that IMF content is mainly determined by the number and size of intramuscular adipocytes (16). Furthermore, IMF is a complex quantitative trait, which is influenced by a variety of regulatory factors, such as gene regulation (17), sex (18), age, or body weight (19), as well as environmental conditions, cell signals, and diet (20). A previous study has shown that IMF plays a key role in many meat quality characteristics and quality (21). For example, IMF can improve meat quality by improving flavor, juiciness, and tenderness (22, 23). Therefore, IMF not only plays a very important role in animal husbandry production but also is closely related to a healthy and desirous human food supply. In recent years, the identification of candidate genes for IMF to improve meat quality has become an important research topic in livestock breeding. A large number of studies have investigated candidate genes affecting IMF content in many species, including cattle (24), pigs (25), sheep (26), and goats (27). Genes, such as PHKG1 (28), MYH3 (20), and PLIN1 (29), have been identified as candidates for regulating IMF content in pigs. However, candidate genes and regulatory mechanisms for IMF content in donkeys are not fully resolved.

With recent developments in proteomic technologies, it has become an increasingly important approach to identify candidate proteins related to meat quality in livestock. A previous study identified 127 proteins with differential abundance associated with IMF in the longissimus dorsi (LD) muscle of pigs between days 120 d and 240 of growth (30). Hou et al. (31) identified proteins from the pig related to postmortem meat quality using a TMT (tandem-mass-tagged)-labeled quantitative proteomic. Similarly, proteomics was used to detect proteins associated with meat quality traits in other species including sheep (32), cattle (33), and chickens (34). Current proteomic studies on the donkey have mainly focused on the identification of proteins associated with donkey milk (35, 36). Few studies on proteins related to the regulation of donkey meat quality have been reported.

In this study, expression profiling of proteins was performed using LD muscle samples with divergent IMF content phenotypes (H and L IMF content groups). We used bioinformatic methods to identify the differentially abundant proteins (DAPs) between the H and L IMF groups. Gene Ontology (GO) and Kyoto Encyclopedia of Genes and Genomes (KEGG) enrichment analysis of the DAPs was conducted. The aim of this study was to identify candidate proteins that influence IMF content in donkeys and to provide a basis for improving the quality of donkey meat.

## Materials and methods

### Ethics statement

All animal procedures described in this study were conducted according to the animal husbandry guidelines of the Shenyang Agricultural University. The studies on these animals were reviewed and approved by the Ethics Committee and Experimental Animal Committee of Shenyang Agriculture University (No. 202006032).

### Sample preparation

All animals used in this study were derived from a population of 30 donkeys described in a previous study (37). These animals were raised under the same environmental conditions. At about 15 months of age, all donkeys are slaughtered in the same abattoir and LD muscle tissue samples were collected for IMF determination and protein extraction. Samples for protein extraction were immediately frozen in liquid nitrogen and then stored at−80°C until use. The Soxhlet extraction method (37) was used to determine the IMF content of the samples.

### Protein extraction and digestion

Protein in the samples was isolated after disruption of the tissue in SDT [4% sodium dodecyl sulfate (SDS), 100 mM Tris-HCl,1 mM DTT, pH 7.6] buffer, with the protein concentration quantified with the bicinchoninic acid (BCA) Protein Assay Kit (Bio-Rad, USA). Protein digestion was performed using trypsin according to the filter-aided sample preparation (FASP) method previously described by Matthias Mann (38). Briefly, protein from each sample was incorporated into SDT [4% SDS, 100 mM dithiothreitol (DTT), 150 mM Tris-HCl pH 8.0] buffer. The detergent, DTT, and other low-molecular-weight components were removed using UA buffer (8 M urea, 150 mM Tris-HCl pH 8.0). Iodoacetamide (IAA), 100 μl of 100 mM IAA in UA buffer, was added to block reduced cysteine residues and incubated in the dark for 30 min. The filter was then washed three times with 100 μl UA buffer and then twice with 100 μl 25 mM NH_4_HCO_3_ buffer. The eluted protein suspension was then digested overnight at 37°C with 4 μg trypsin (Promega) in 40 μl of 25 mM NH_4_HCO_3_ buffer and the resulting peptides were collected as filtrate. Digested peptides for each sample were desalted on C_18_ cartridges, concentrated by vacuum centrifugation, and reconstituted in 40 μl of 0.1% (v/v) formic acid.

### Tandem-mass-tag labeling

Peptides from the three high (H1, H2, and H3) and three low (L1, L2, and L3) IMF content donkey samples were labeled with 126, 127, 128, 129, 130, and 131 isotope tags, respectively, using TMT reagents according to the manufacturer's instructions (Thermo Scientific, Waltham, USA).

### Liquid chromatography–mass spectrometry analysis

Liquid chromatography–mass spectrometry (LC–MS/MS) analysis was conducted on a Q Exactive mass spectrometer (Thermo Scientific, Waltham, USA). MS data were obtained using a data-dependent top 10 method dynamically choosing the most abundant precursor ions from the survey scan (300–1,800 m/z) for high-energy collisional dissociation (HCD) fragmentation. The automatic gain control (AGC) target was 3 × 10^6^ and the maximum injection time was 10 ms. The dynamic exclusion duration was 40.0 s. Survey scans were obtained at a resolution of 70,000 at m/z 200 and resolution for HCD spectra was set to 17,500 at m/z 200, and isolation width was 2 m/z. The normalized collision energy was 30 eV and the underfill ratio was 0.1%.

### Identification and quantitation of proteins

The MS raw data for each sample were searched against the donkey UniProt database using the MASCOT engine (Matrix Science, London, UK; version 2.2) embedded into Proteome Discoverer 1.4 software for protein identification and quantitation. Proteins with fold change (FC) >1.2 or <0.833 and *p* < 0.05 were considered to be significantly DAPs.

### Gene ontology and KEGG pathway enrichment analysis of DAPs

The gene ontology (GO) term annotation of selected DAPs was performed using Blast2GO software (39, 40). The DAPs were blasted against the Kyoto Encyclopedia of Genes and Genomes (KEGG) database (http://geneontology.org/) to retrieve their KEGG orthology identifications, which were subsequently mapped to the pathways in KEGG (41). GO and KEGG enrichment was analyzed using the Fisher's exact test. GO and KEGG terms with *p* < 0.05 were considered significantly enriched.

### Protein–protein interactions analysis

For protein–protein interactions, we used STRING v11.5 (42) to predict and visualize networks according to default parameters. Protein interactions were illustrated using Cytoscape software (43).

## Results

### Characterization of identified proteins

**Figure 1:**
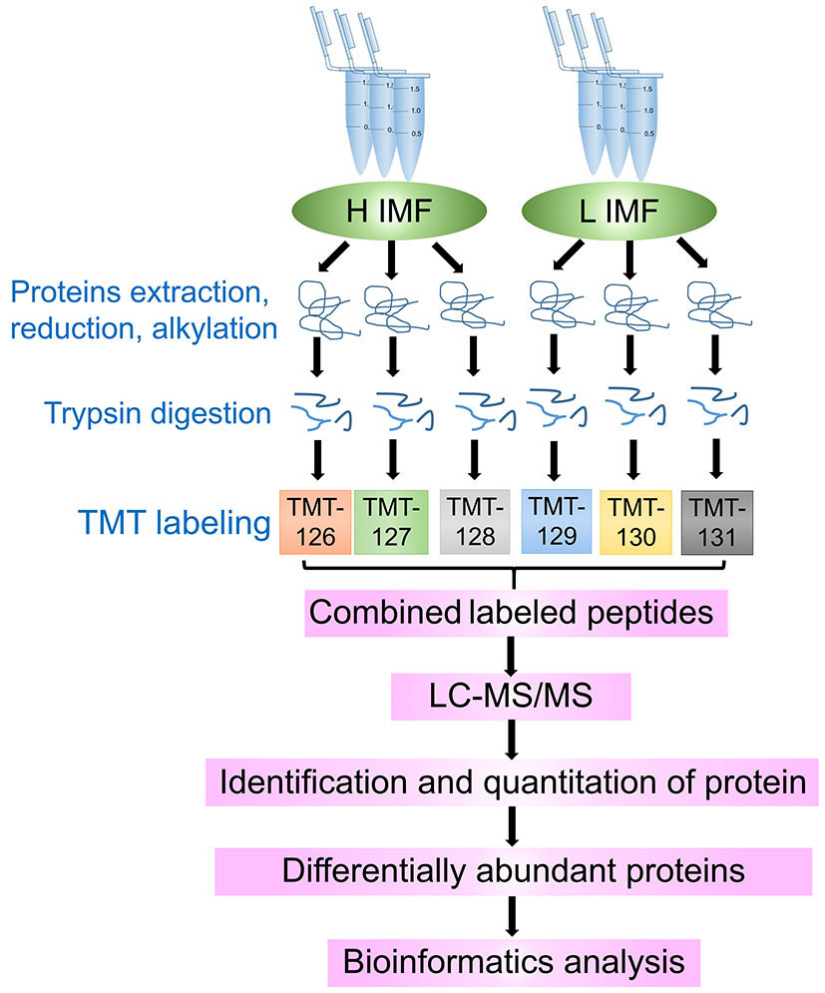
Schematic protocol for the identification of longissimus dorsi muscle proteins from tissue samples with high (H) and low (L) intramuscular fat (IMF) content using tandem-mass-tagged (TMT)-labeled quantitative proteomics. The H and L IMF samples were in triplicate, and each sample used a different TMT label.

**Figure 2:**
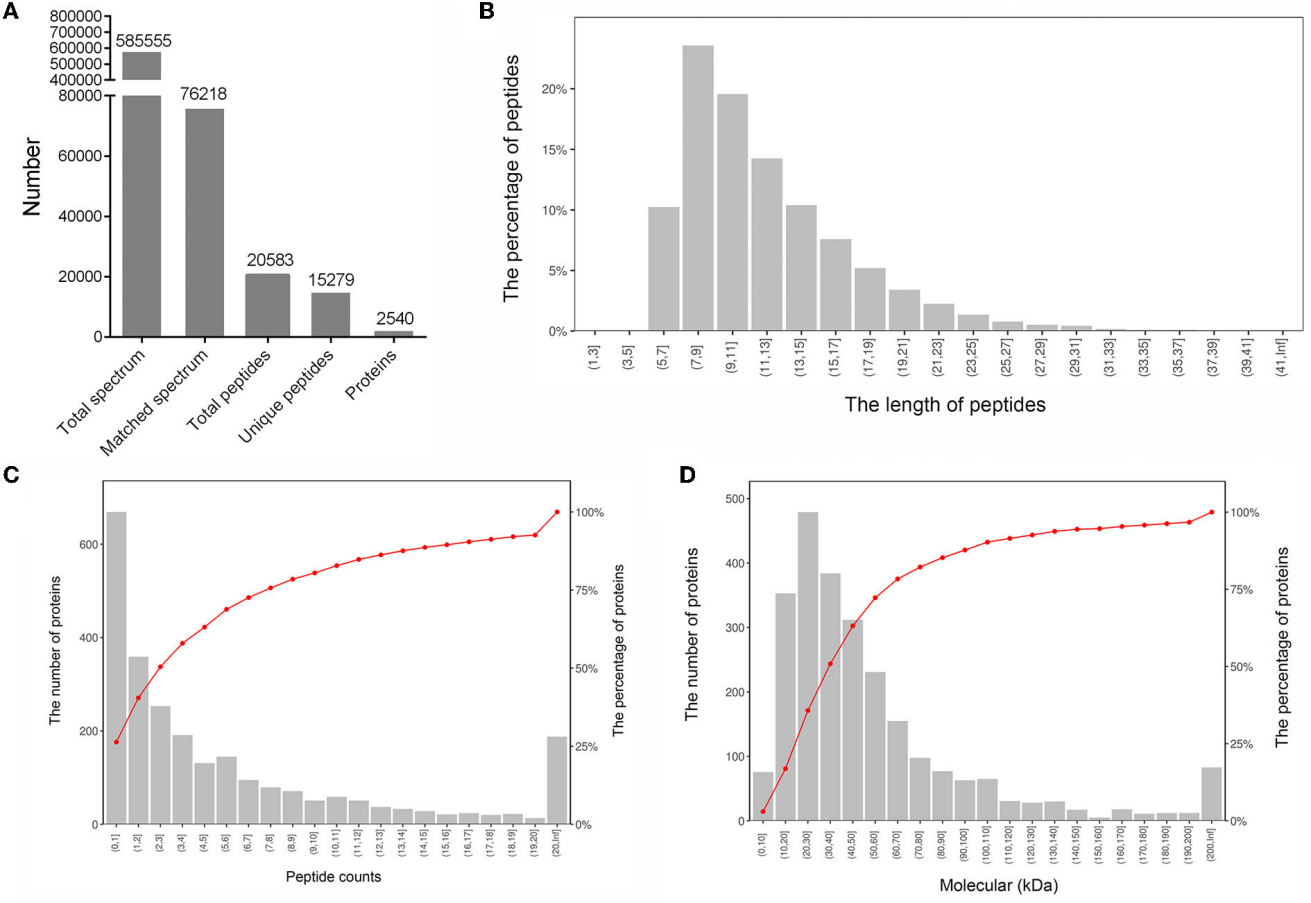
Characterization of proteins from six longissimus dorsi muscle samples. **(A)** Overview information of identified proteins in this study. **(B)** Distribution of the lengths of the identified peptides. **(C)** Distribution of the numbers of identified proteins containing different numbers of peptides. **(D)** Distribution of the molecular weights of the identified proteins.

We selected three LD muscle samples with high (H1, H2, and H3) and three with low (L1, L2, and L3) IMF content from a population of 30 individuals. Statistical analysis of the data showed that IMF content was significantly different between H and L samples (3.04 ± 0.12%, 6.39 ± 0.47%; *p* < 0.01). We further characterized the protein abundance profiles of the six samples using the TMT-labeled proteomic approach. The entire proteomic experimental flow for this study is shown in Figure 1. A total of 585,555 spectra were detected from the six LD muscle samples by LC–MS/MS analysis (Figure 2A). From this, we identified a total of 20,583 peptides, of which 15,279 were unique, corresponding to 2,540 distinct proteins (Figure 2A; Supplementary Table S1). A statistical analysis of the lengths of the identified peptide was conducted, which showed that the peptides were mainly between 5 and 15 amino acids with peptides of 7 and 9 amino acids in length being the highest (Figure 2B). Further analyses of the numbers of peptides identified in the proteins showed that about 50% of the proteins contained 1–3 identified peptides (Figure 2C). The molecular weights of the identified proteins indicated that most of these proteins have molecular weights between 10 and 70 kDa (Figure 2D).

### Identification of DAPs between high and low IMF groups

**Figure 3:**
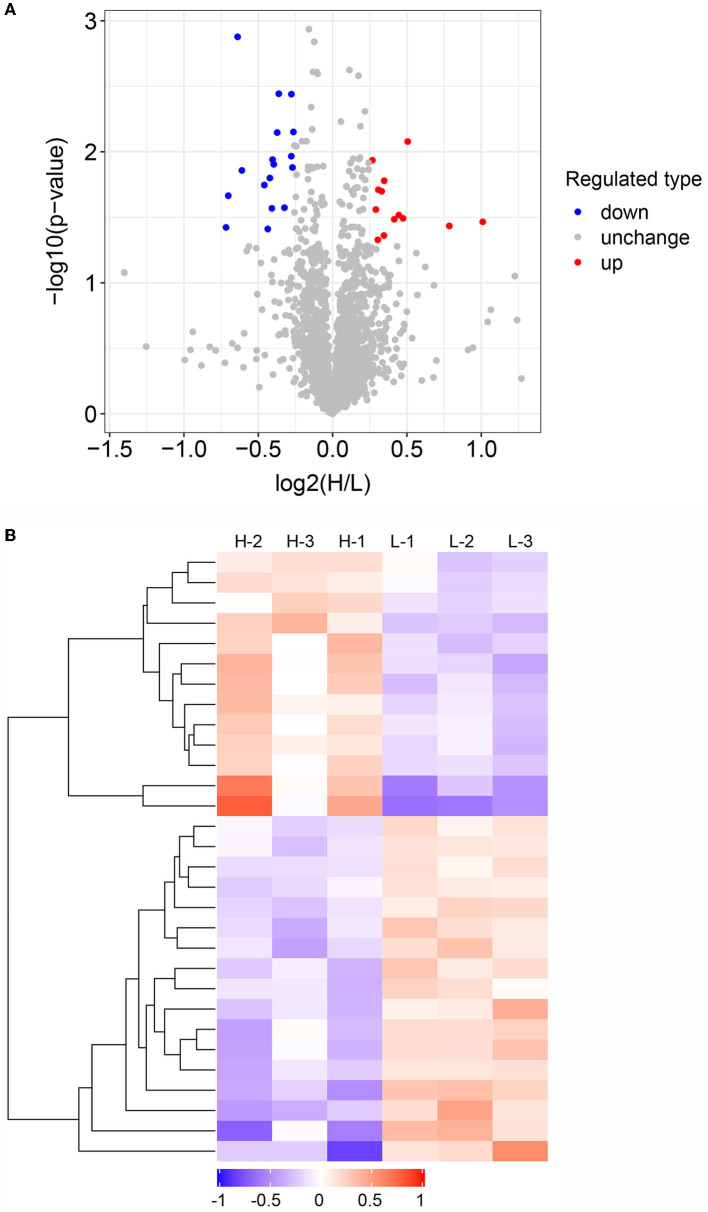
Analysis of differentially abundant proteins (DAPs) between high and low intramuscular fat (IMF) groups. **(A)** Volcano plot showing DAPs between high and low IMF groups. Red, gray, and blue dots represent upregulated, unchanged, and downregulated proteins, respectively. **(B)** Heatmap showing the abundance patterns of the DAPs between the three high and three low IMF samples.

To further explore candidate proteins associated with IMF deposition in donkeys, we analyzed the differentially abundant proteins in the high and low IMF content groups. A total of 30 DAPs were identified between the H and L IMF groups, with 13 upregulated and 17 downregulated in the H IMF group (Figure 3A). Summary information on these proteins is listed in Supplementary Table S2. The top upregulated DAP is MAP4K4, while the top downregulated DAP is Rho-associated protein kinase 2 (ROCK2) (Supplementary Table S2). We also examined the abundance patterns of these DAPs in the six samples, which showed clear abundance differences between the high and low IMF group samples (Figure 3B).

### Gene ontology and KEGG enrichment analysis of DAPs

**Figure 4:**
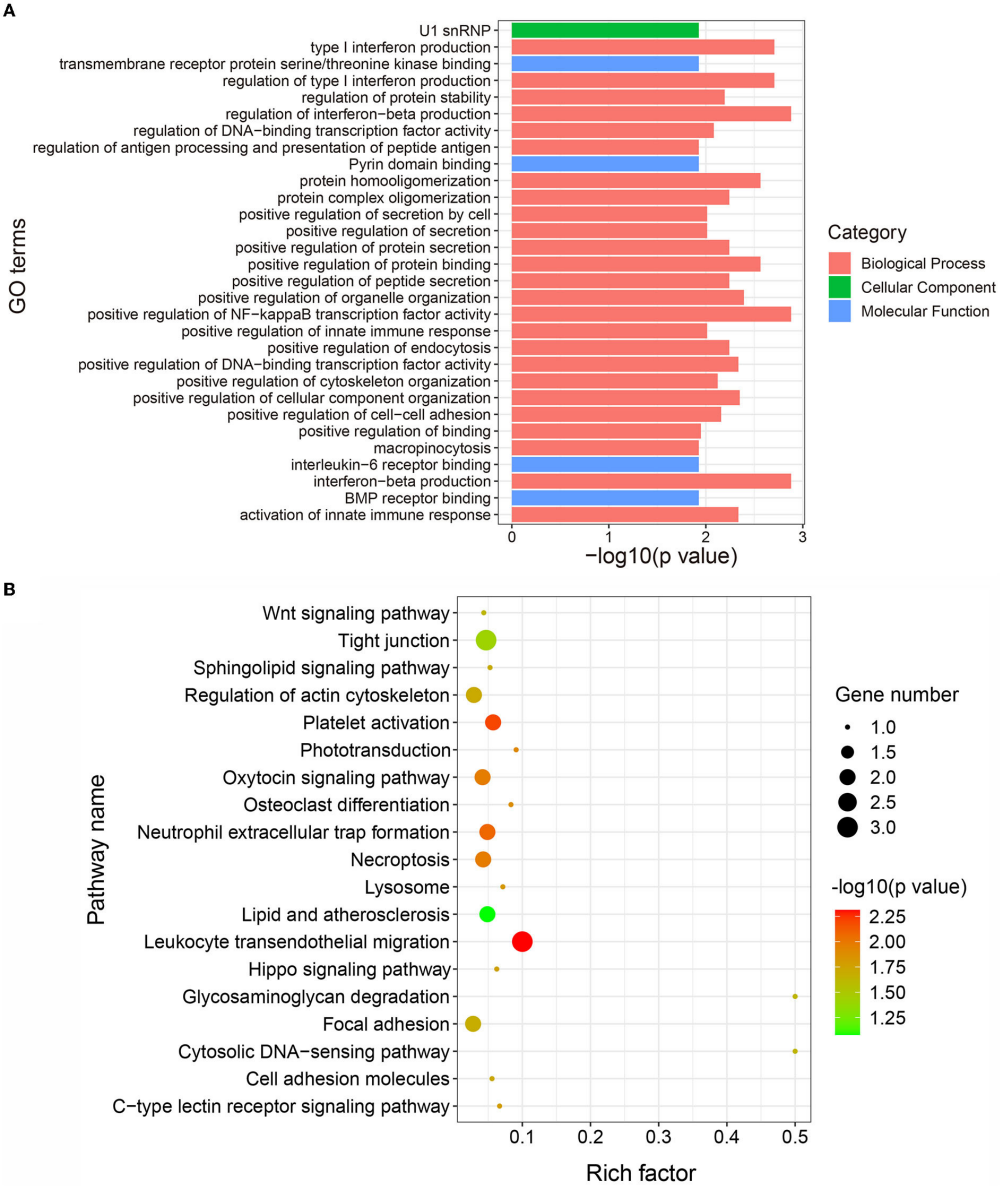
Gene ontology (GO) and Kyoto Encyclopedia of Genes and Genomes (KEGG) functional enrichment analysis of differentially abundant proteins (DAPs). **(A)** Top 30 significantly enriched GO terms. The X-axis represents the –log10 (*p* value) value of the GO terms and the Y-axis represents the GO term name. **(B)** Significantly enriched pathways of DAPs. The X-axis represents the rich factor and the Y-axis represents the pathway. The size and color of the bubbles represent the number of proteins enriched in the pathway and enrichment significance, respectively.

To explore the potential biological functions of the DAPs in IMF deposition, we performed GO and KEGG functional enrichment analyses of the DAPs. Here, 227, 27, and 24 GO terms were found to be significantly enriched in biological process, cellular component, and molecular function, respectively (Supplementary Table S3). The top 30 significantly enriched GO terms are shown in Figure 4A. This data suggests that the DAPs are primarily involved in bone morphogenetic protein (BMP) receptor binding, which is associated with lipid metabolism. The KEGG enrichment analysis suggested that the DAPs are significantly enriched in 19 pathways (Figure 4B). Interestingly, some of these KEGG participate in the lipogenic processes, for instance, the Wnt signaling and Hippo signaling pathways. Further, a DAP, ROCK2, is significantly enriched in the Wnt signaling pathway. These results suggest that the enrichment of DAPs in these pathways may be closely related to the lipogenic process.

### Protein–protein interactions analysis for DAPs

**Figure 5:**
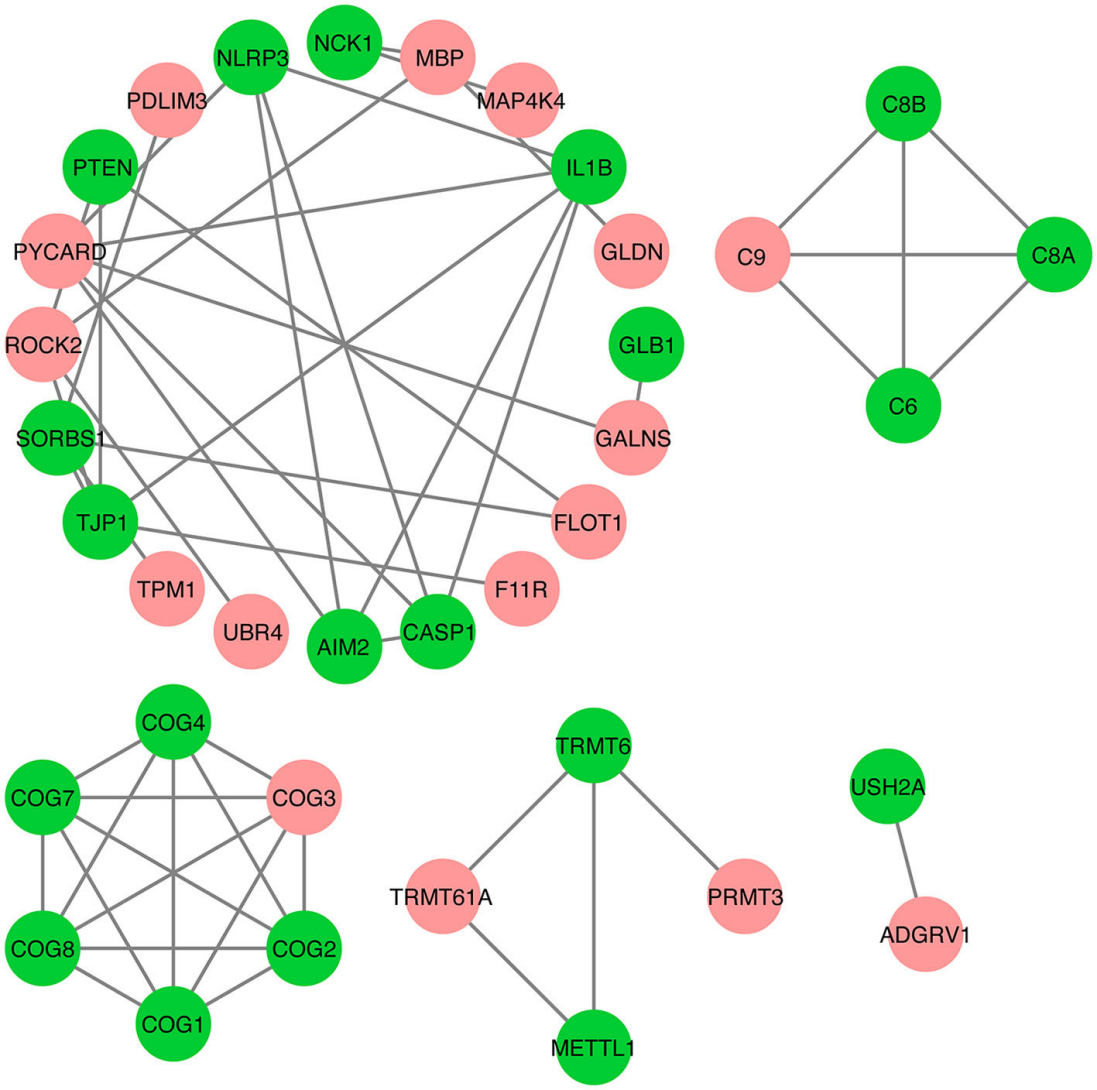
Protein–protein interactions analysis for differentially abundant proteins (DAPs). The red nodes represent DAPs, while the green nodes represent other proteins that interact with DAPs. The edge represents interactions between proteins.

Next, we examined protein–protein interactions of the 30 DAPs to better understand IMF deposition. A protein–protein interaction analysis of the DAPs was conducted based on the STRING database, which contains details functional relationships between proteins, thus allowing predictions on the functional impact of changing protein abundance (44). The results of this analysis showed that 16 DAPs are involved in a protein–protein interaction network (Figure 5). For example, a DAP, ROCK2, interacts with UBR4, TJP1, MBP, and PTEN. In addition, the ADGRV1 and USH2A proteins interact with each other.

## Discussion

In recent years, consumers have become increasingly interested in donkey meat, with China becoming the world's largest consumer of this meat (45). IMF plays a vital role in the quality of livestock meat, therefore, the identification of candidate proteins affecting IMF is essential for improving meat quality. Previous studies have applied proteomics to identify DAPs of IMF in other species including pigs (30) and goats (46). However, there is a paucity of research on candidate proteins for quality traits in donkey meat. In this study, we identified 30 proteins that were differentially abundant between LD muscle samples with high and low IMF content based on proteomic techniques. Our results suggest that the identified DAPs are candidates for influencing IMF content in donkey meat.

Further, of these DAPs, many are associated with the adipogenic processes. For example, ROCK2, a downregulated protein, inhibits adipogenesis based on knockdown experiments (47). Protein interaction analysis revealed that ROCK2 potentially binds UBR4, TJP1, MBP, and PTEN proteins. These results suggest that the ROCK2 protein might act as a suppressor of IMF deposition in the donkey by binding these proteins. Ahbara et al. (48) revealed the SPAG8 gene is a candidate for growth traits and adipogenesis in sheep. A previous study identified the RPL27A gene as a candidate gene for bovine marbling, with an single nucleotide polymorphism (SNP) in its promoter used as a molecular marker for bovine marbling (49). Tu et al. (50) found that a high-fat diet increases the expression level of TPM1. Another interesting finding is that PRMT3 acts as a co-transcription factor by translocating into the nucleus and binding to LXRα to regulate downstream gene expression, thereby promoting lipogenesis (51). These proteins (i.e., SPAG8, RPL27A, TPM1, MBP, and PRMT3) were identified as DAPs between the H and L IMF groups in this study, suggesting that they are important regulators of IMF. However, the mechanism of action of these DAPs in IMF deposition in donkeys needs to be further demonstrated.

Our study identified a number of GO terms related to the lipogenic process based on enrichment analysis of the DAPs, including tRNA methylation and BMP receptor binding. Zhao et al. (52) showed that RNA methylation has a crucial role and is required for adipose differentiation. In addition, it has been shown that tRNA methylation levels regulate the translation initiation of genes and thus affect protein synthesis (53). These data suggest that some DAPs may regulate IMF deposition by affecting tRNA methylation. BMP signaling is essential for the differentiation of mesenchymal stem cells (MSCs) into the adipocyte lineage (54). Jung et al. (55) found that plasma BMP2 levels were positively correlated with IMF content in steers, suggesting that BMP signaling contributes to meat quality. In addition, a GO term, positive regulation of c-Jun N-terminal kinase (JNK) cascade, was significantly enriched in this study. A previous study demonstrated that JNK signaling inhibits adipose differentiation by suppressing peroxisome proliferator-activated receptor-gamma (PPARγ) and fatty acid binding protein 4 (AP2) expression (56). Taken together, these DAPs may regulate the deposition of IMF through an association with adipogenesis.

In addition, DAPs were enriched in many key signaling pathways (e.g., Wnt and Hippo signaling pathways) involved in the adipogenic process. The Wnt signaling pathway is a highly conserved critical factor in animals that negatively regulates adipose differentiation (57). Mechanistically, Wnt signaling inhibits adipogenesis by suppressing the expression of PPARγ and CCAAT/enhancer-binding protein alpha (C/EBP)α (58). Activation of Wnt signaling, by transgenic overexpression of Wnt10, in mice leads to significantly reduced adipose tissue weight (59). A downregulated protein identified in our study, ROCK2, is significantly enriched in the Wnt signaling pathway, suggesting that it inhibits IMF deposition by mediating the Wnt signaling pathway. The Hippo signaling pathway was also enriched, and it has been shown to be involved in adipocyte proliferation and differentiation in animals (60, 61). In Hippo signaling pathway, serine/threonine kinase 24 (Ste20) family kinases mammalian STE20-like kinase (MST1/2) activate large tumor suppressor (LATS) kinases through phosphorylation, which in turn allows phosphorylation of yes-associated protein (YAP) protein and facilitates the binding of YAP to 14-3-3 proteins (62). Park et al. (63) found that MST1/2 promotes the differentiation of 3T3-L1 cells through the activation of PPARγ. Deng et al. (61) demonstrated that YAP1 inhibits the differentiation of ovine adipocytes by affecting PPARG and RXR alpha levels. These results suggest that the Hippo signaling pathway is implicated in the deposition of IMF in the donkey. However, how DAPs involved in Hippo signaling regulate the deposition of IMF in donkeys still needs to be further explored.

## Conclusion

In summary, we identified 30 candidate proteins that might affect IMF content in the LD muscle of donkeys. We provide evidence that some of the DAPs affect IMF deposition as they are involved in adipogenic functions or signaling pathways in other animals. Our data provide evidence for the role of these proteins in IMF content in the donkey and provide new insights into the molecular mechanisms of the regulation of IMF deposition.

## Data availability statement

The original contributions presented in the study are included in the article/Supplementary material, further inquiries can be directed to the corresponding author/s.

## Ethics statement

The animal study was reviewed and approved by the Ethics Committee and Experimental Animal Committee of Shenyang Agriculture University. Written informed consent was obtained from the owners for the participation of their animals in this study.

## Author contributions

BL and SZha conceived and designed this study. YH, YQ, and XT performed the experiments. ZY, JC, SZho, and RZ analyzed the data. XT and BL drafted the manuscript. DI and BL revised the manuscript.

## Funding

This research was supported by grants from the National Natural Science Foundation of China (No. 32002147), the China Postdoctoral Science Foundation (No. 2021MD703855), the Educational Department of Liaoning Province (No. LJKZ0671), the Natural Science Foundation of Liaoning Province (No. 2021-BS-140), the Science and Technology Plan Project of Shenyang City (Nos. 21-116-3-40 and 21-110-3-10), the Organization Department of Liaoning Provincial Committee of China (No. XLYC1907018), and the Shenyang Agricultural University Research Start-up Funding (No. 880418062).

## Conflict of interest

The authors declare that the research was conducted in the absence of any commercial or financial relationships that could be construed as a potential conflict of interest.

## Publisher's note

All claims expressed in this article are solely those of the authors and do not necessarily represent those of their affiliated organizations, or those of the publisher, the editors and the reviewers. Any product that may be evaluated in this article, or claim that may be made by its manufacturer, is not guaranteed or endorsed by the publisher.

## References

[1] Orlando L. Equids. Curr Biol. (2015) 25:R973–978. 10.1016/j.cub.2015.09.005 26485367

[2] Shi T Hu W Hou H Zhao Z Shang M Zhang L. Identification and comparative analysis of long non-coding RNA in the skeletal muscle of two Dezhou Donkey strains. Genes (Basel). (2020) 11:508. 10.3390/genes11050508 32375413 PMC7288655

[3] Polidori P Vincenzetti S Cavallucci C Beghelli D. Quality of donkey meat and carcass characteristics. Meat Sci. (2008) 80:1222–4. 10.1016/j.meatsci.2008.05.027 22063861

[4] De Palo P Maggiolino A Milella P Centoducati N Papaleo A Tateo A. Artificial suckling in Martina Franca donkey foals: effect on in vivo performances and carcass composition. Trop Anim Health Prod. (2016) 48:167–73. 10.1007/s11250-015-0940-2 26510946

[5] De Palo P Tateo A Maggiolino A Marino R Ceci E Nisi A. Martina Franca donkey meat quality: influence of slaughter age and suckling technique. Meat Sci. (2017) 134:128–34. 10.1016/j.meatsci.2017.07.025 28783609

[6] Polidori P Pucciarelli S Ariani A Polzonetti V Vincenzetti S A. comparison of the carcass and meat quality of Martina Franca donkey foals aged 8 or 12 months. Meat Sci. (2015) 106:6–10. 10.1016/j.meatsci.2015.03.018 25863189

[7] Trinchese G Cavaliere G De Filippo C Aceto S Prisco M Chun JT. Human milk and donkey milk, compared to cow milk, reduce inflammatory mediators and modulate glucose and lipid metabolism, acting on mitochondrial function and oleylethanolamide levels in rat skeletal muscle. Front Physiol. (2018) 9:32. 10.3389/fphys.2018.00032 29472867 PMC5810302

[8] Zhang X Li H Yu J Zhou X Ji C Wu S. Label-free based comparative proteomic analysis of whey proteins between different milk yields of Dezhou donkey. Biochem Biophys Res Commun. (2019) 508:237–42. 10.1016/j.bbrc.2018.11.130 30482389

[9] Lanza M Landi C Scerra M Galofaro V Pennisi P. Meat quality and intramuscular fatty acid composition of Sanfratellano and Haflinger foals. Meat Sci. (2009) 81:142–7. 10.1016/j.meatsci.2008.07.008 22063974

[10] Lorenzo JM Sarries MV Tateo A Polidori P Franco D Lanza M. Carcass characteristics, meat quality and nutritional value of horsemeat: a review. Meat Sci. (2014) 96:1478–88. 10.1016/j.meatsci.2013.12.006 24423453

[11] Seyiti S Kelimu A. Donkey industry in China: current aspects, suggestions and future challenges. J Equine Vet Sci. (2021) 102:103642. 10.1016/j.jevs.2021.103642 34119208

[12] Polidori P Vincenzetti S Pucciarelli S Polzonetti V. Comparison of carcass and meat quality obtained from mule and donkey. Animals-Basel. (2020) 10:1620. 10.3390/ani10091620 32927781 PMC7552182

[13] Listrat A Lebret B Louveau I Astruc T Bonnet M Lefaucheur L. How muscle structure and composition influence meat and flesh quality. ScientificWorldJournal. (2016) 2016:3182746. 10.1155/2016/3182746 27022618 PMC4789028

[14] da Costa AS Pires VM Fontes CM Mestre Prates JA. Expression of genes controlling fat deposition in two genetically diverse beef cattle breeds fed high or low silage diets. BMC Vet Res. (2013) 9:118. 10.1186/1746-6148-9-118 23767408 PMC3691746

[15] Silva DBD Fonseca LFS Pinheiro DG Muniz MMM Magalhaes AFB Baldi F. Prediction of hub genes associated with intramuscular fat content in Nelore cattle. BMC Genom. (2019) 20:1–12. 10.1186/s12864-019-5904-x 31238883 PMC6591902

[16] Li X Fu X Yang G Du M. Review: Enhancing intramuscular fat development via targeting fibro-adipogenic progenitor cells in meat animals. Animal. (2020) 14:312–21. 10.1017/S175173111900209X 31581971

[17] Plastow GS Carrion D Gil M Garcia-Regueiro JA Gispert M Oliver MA. Quality pork genes and meat production. Meat Sci. (2005) 70:409–21. 10.1016/j.meatsci.2004.06.025 22063741

[18] Skrlep M Batorek N Bonneau M Prevolnik M Kubale V Candek-Potokar M. Effect of immunocastration in group-housed commercial fattening pigs on reproductive organs, malodorous compounds, carcass and meat quality. Czech J Anim Sci. (2012) 57:290–9. 10.17221/5964-CJAS

[19] Tyra M Ropka-Molik K Terman A Piorkowska K Oczkowicz M Bereta A. Association between subcutaneous and intramuscular fat content in porcine ham and loin depending on age, breed and FABP3 and LEPR genes transcript abundance. Mol Biol Rep. (2013) 40:2301–8. 10.1007/s11033-012-2311-7 23192618 PMC3563946

[20] Cho IC Park HB Ahn JS Han SH Lee JB Lim HT. A functional regulatory variant of MYH3 influences muscle fiber-type composition and intramuscular fat content in pigs. PLoS Genet. (2019) 15:e1008279. 10.1371/journal.pgen.1008279 31603892 PMC6788688

[21] Gao SZ Zhao SM. Physiology, affecting factors and strategies for control of pig meat intramuscular fat. Recent Pat Food Nutr Agric. (2009) 1:59–74. 10.2174/2212798410901010059 20653527

[22] Hausman GJ Dodson MV Ajuwon K Azain M Barnes KM Guan LL. Board-invited review: the biology and regulation of preadipocytes and adipocytes in meat animals. J Anim Sci. (2009) 87:1218–46. 10.2527/jas.2008-1427 18849378

[23] Li QQ Huang ZY Zhao WJ Li MX Li CC. Transcriptome analysis reveals long intergenic non-coding RNAs contributed to intramuscular fat content differences between Yorkshire and Wei pigs. Int J Mol Sci. (2020) 21:1732. 10.3390/ijms21051732 32138348 PMC7084294

[24] Ochsner KP MacNeil MD Lewis RM Spangler ML. Economic selection index development for Beefmaster cattle I: terminal breeding objective. J Anim Sci. (2017) 95:1063–70. 10.2527/jas.2016.1231 28380518

[25] Gao Y Zhang R Hu X Li N. Application of genomic technologies to the improvement of meat quality of farm animals. Meat Sci. (2007) 77:36–45. 10.1016/j.meatsci.2007.03.026 22061394

[26] Mortimer SI van der Werf JHJ Jacob RH Hopkins DL Pannier L Pearce KL. Genetic parameters for meat quality traits of Australian lamb meat. Meat Sci. (2014) 96:1016–24. 10.1016/j.meatsci.2013.09.007 24084607

[27] Pena F Juarez M Bonvillani A Garcia P Polvillo O Domenech V. Muscle and genotype effects on fatty acid composition of goat kid intramuscular fat. Ital J Anim Sci. (2011) 10:212–6. 10.4081/ijas.2011.e40

[28] Ma JW Yang J Zhou LS Ren J Liu XX Zhang H. A splice mutation in the PHKG1 gene causes high glycogen content and low meat quality in pig skeletal muscle. PLoS Genet. (2014) 10:e1004710. 10.1371/journal.pgen.1004710 25340394 PMC4207639

[29] Li BJ Weng QN Dong C Zhang ZK Li RY Liu JG. A key gene, PLIN1, can affect porcine intramuscular fat content based on transcriptome analysis. Genes. (2018) 9:194. 10.3390/genes9040194 29617344 PMC5924536

[30] Ma C Wang WW Wang YD Sun Y Kang L Zhang Q. TMT-labeled quantitative proteomic analyses on the longissimus dorsi to identify the proteins underlying intramuscular fat content in pigs. J Proteomics. (2020) 213:103630. 10.1016/j.jprot.2019.103630 31881348

[31] Hou XH Liu QF Meng QS Wang LG Yan H Zhang LC. TMT-based quantitative proteomic analysis of porcine muscle associated with postmortem meat quality. Food Chem. (2020) 328:127133. 10.1016/j.foodchem.2020.127133 32480263

[32] Ma DY Yu QQ Hedrick VE Cooper BR Sobreira TJP Oh JH. Proteomic and metabolomic profiling reveals the involvement of apoptosis in meat quality characteristics of ovine M. longissimus from different callipyge genotypes. Meat Sci. (2020) 166:108140. 10.1016/j.meatsci.2020.108140 32298943

[33] Poleti MD Regitano LCA Souza G Cesar ASM Simas RC Silva-Vignato B. Longissimus dorsi muscle label-free quantitative proteomic reveals biological mechanisms associated with intramuscular fat deposition. J Proteomics. (2018) 179:30–41. 10.1016/j.jprot.2018.02.028 29510239

[34] Zhang J Cao J Geng A Wang H Chu Q Yang L. Comprehensive proteomic characterization of the pectoralis major at three chronological ages in Beijing-you chicken. Front Physiol. (2021) 12:658711. 10.3389/fphys.2021.658711 33815156 PMC8012914

[35] Li W Li M Cao X Han H Kong F Yue X. Comparative analysis of whey proteins in donkey colostrum and mature milk using quantitative proteomics. Food Res Int. (2020) 127:108741. 10.1016/j.foodres.2019.108741 31882075

[36] Zhang X Jiang B Ji C Li H Yang L Jiang G. Quantitative label-free proteomic analysis of milk fat globule membrane in donkey and human milk. Front Nutr. (2021) 8:670099. 10.3389/fnut.2021.670099 34239890 PMC8258387

[37] Li B Feng C Zhu S Zhang J Irwin DM Zhang X. Identification of candidate circular RNAs underlying intramuscular fat content in the donkey. Front Genet. (2020) 11:587559. 10.3389/fgene.2020.587559 33424924 PMC7793956

[38] Wisniewski JR Zougman A Nagaraj N Mann M. Universal sample preparation method for proteome analysis. Nat Methods. (2009) 6:359–62. 10.1038/nmeth.1322 19377485

[39] Ashburner M Ball CA Blake JA Botstein D Butler H Cherry JM. Gene ontology: tool for the unification of biology. Gene Ontol Consortium Nat Genet. (2000) 25:25–9. 10.1038/75556 10802651 PMC3037419

[40] Gotz S Garcia-Gomez JM Terol J Williams TD Nagaraj SH Nueda MJ. High-throughput functional annotation and data mining with the Blast2GO suite. Nucleic Acids Res. (2008) 36:3420–35. 10.1093/nar/gkn176 18445632 PMC2425479

[41] Kanehisa M Goto S Sato Y Furumichi M Tanabe M KEGG. for integration and interpretation of large-scale molecular data sets. Nucleic Acids Res. (2012) 40:D109–114. 10.1093/nar/gkr988 22080510 PMC3245020

[42] Szklarczyk D Gable AL Lyon D Junge A Wyder S Huerta-Cepas J. STRING v11: protein-protein association networks with increased coverage, supporting functional discovery in genome-wide experimental datasets. Nucleic Acids Res. (2019) 47:D607–13. 10.1093/nar/gky1131 30476243 PMC6323986

[43] Shannon P Markiel A Ozier O Baliga NS Wang JT Ramage D. Cytoscape: a software environment for integrated models of biomolecular interaction networks. Genome Res. (2003) 13:2498–504. 10.1101/gr.1239303 14597658 PMC403769

[44] Liu G Wong L Chua HN. Complex discovery from weighted PPI networks. Bioinformatics. (2009) 25:1891–7. 10.1093/bioinformatics/btp311 19435747

[45] Polidori P Cammertoni N Santini G Klimanova Y Zhang JJ Vincenzetti S. Effects of donkeys rearing system on performance indices, carcass, and meat quality. Foods. (2021) 10:3119. 10.3390/foods10123119 34945670 PMC8701087

[46] Du Y Wang Y Xu Q Zhu J Lin Y. TMT-based quantitative proteomics analysis reveals the key proteins related with the differentiation process of goat intramuscular adipocytes. BMC Genom. (2021) 22:417. 10.1186/s12864-021-07730-y 34090334 PMC8180059

[47] Noguchi M Hosoda K Fujikura J Fujimoto M Iwakura H Tomita T. Genetic and pharmacological inhibition of Rho-associated kinase II enhances adipogenesis. J Biol Chem. (2007) 282:29574. 10.1074/jbc.M705972200 17681946

[48] Ahbara A Bahbahani H Almathen F Al Abri M Agoub MO Abeba A. Genome-wide variation, candidate regions and genes associated with fat deposition and tail morphology in Ethiopian indigenous sheep. Front Genet. (2018) 9:699. 10.3389/fgene.2018.00699 30687385 PMC6334744

[49] Yamada T Sasaki S Sukegawa S Miyake T Fujita T Kose H. Association of a single nucleotide polymorphism in ribosomal protein L27a gene with marbling in Japanese Black beef cattle. Anim Sci J. (2009) 80:631–5. 10.1111/j.1740-0929.2009.00688.x 20163651

[50] Tu ZL Yu B Huang DY Ojha R Zhou SK An HD. Proteomic analysis and comparison of intra and extracranial cerebral atherosclerosis responses to hyperlipidemia in rabbits. Mol Med Rep. (2017) 16:2347–54. 10.3892/mmr.2017.6869 28677755 PMC5548028

[51] Kim DI Park MJ Lim SK Park JI Yoon KC Han HJ. PRMT3 regulates hepatic lipogenesis through direct interaction with LXRα. Diabetes. (2015) 64:60. 10.2337/db13-1394 25187371

[52] Zhao X Yang Y Sun BF Yue S Xin Y Xiao W. FTO-dependent demethylation of N6-methyladenosine regulates mRNA splicing and is required for adipogenesis. Cell Res. 24:1403–19. 10.1038/cr.2014.151 25412662 PMC4260349

[53] Liu F Clark W Luo G Wang X He CJC. ALKBH1-mediated tRNA demethylation regulates translation. (2016) 167:233–47. 10.1016/j.cell.2016.11.045 27984735

[54] Huang H Song TJ Li X Hu L He Q Liu M. BMP signaling pathway is required for commitment of C3H10T1/2 pluripotent stem cells to the adipocyte lineage. Proc Natl Acad Sci USA. (2009) 106:12670–5. 10.1073/pnas.0906266106 19620713 PMC2722335

[55] Jung DS Baik Baik M Up-regulation of bone morphogenetic protein and its signaling molecules following castration of bulls and their association with intramuscular fat content in Korean cattle. Sci Rep-UK. (2019) 9:1–7. 10.1038/s41598-019-56439-2 31875043 PMC6930278

[56] Sanyal A Naumann J Hoffmann LS Chabowska-Kita A Ehrlund A Schlitzer A. Interplay between obesity-induced inflammation and cGMP signaling in white adipose tissue. Cell Rep. (2017) 18:225. 10.1016/j.celrep.2016.12.028 28052251

[57] Ross SE Hemati N Longo KA Bennett CN Lucas PC Erickson RL. Inhibition of adipogenesis by Wnt signaling. Science. (2000) 289:950–3. 10.1126/science.289.5481.950 10937998

[58] Rosen ED MacDougald OA. Adipocyte differentiation from the inside out. Nat Rev Mol Cell Biol. (2006) 7:885–96. 10.1038/nrm2066 17139329

[59] Wright WS Longo KA Dolinsky VW Gerin I Kang S Bennett CN. Wnt10b inhibits obesity in ob/ob and agouti mice. Diabetes. (2007) 56:295–303. 10.2337/db06-1339 17259372

[60] Hong JH Hwang ES McManus MT Amsterdam A Tian Y Kalmukova RTAZ a a transcriptional modulator of mesenchymal stem cell differentiation. J Sci. (2005) 309:1074–8. 10.1126/science.1110955 16099986

[61] Deng K Ren C Fan Y Pang J Zhang G Zhang Y. YAP1 regulates PPARG and RXR alpha expression to affect the proliferation and differentiation of ovine preadipocyte. J Cell Biochem. (2019) 120:19578–89. 10.1002/jcb.29265 31297878

[62] Ibar C Irvine KD. DC integration of hippo-YAP signaling with metabolism. Dev Cell. (2020) 54:256–67. 10.1016/j.devcel.2020.06.025 32693058 PMC7373816

[63] Park BH Kim DS Won GW Jeon HJ Oh BC Lee Y. Mammalian Ste20-like kinase and SAV1 promote 3T3-L1 adipocyte differentiation by activation of PPARγ. PLoS ONE. (2012) 7:e30983. 10.1371/journal.pone.0030983 22292086 PMC3266932

